# Accelerators for improved health among adolescent mothers in South Africa: HIV and violence prevention, sexual reproductive health and education success

**DOI:** 10.1136/bmjgh-2024-017614

**Published:** 2025-06-02

**Authors:** Lucie Cluver, Janina Jochim, Lulama Sidloyi, Alice Armstrong, Laurie Gulaid, Bolade Banougnin, Bongiwe Saliwe, Kathryn Steventon Roberts, Mildred Thabeng, Kristen de Graaf, Elona Toska

**Affiliations:** 1 Department of Social Policy and Intervention, University of Oxford, Oxford, Oxfordshire, UK; 2 Department of Psychiatry, University of Cape Town, Rondebosch, South Africa; 3 Oxford Research South Africa, Oxford, UK; 4 UNICEF Eastern and Southern Africa Regional Office, Nairobi, Kenya; 5 Centre for Social Science Research, University of Cape Town, Rondebosch, South Africa; 6 West and Central Africa Office, UNFPA Senegal, Dakar, Senegal; 7 Institute for Global Health, University College London, London, UK; 8 University of Oxford, Oxford, UK; 9 Centre for Social Science Research, University of Cape Town, Cape Town, Western Cape, South Africa

**Keywords:** Epidemiology, HIV

## Abstract

**Background:**

30% of girls in Africa are mothers, facing high risk for negative health and educational outcomes. We aimed to identify services with the potential to reduce multiple simultaneous risks for adolescent mothers, described by the UN as ‘development accelerators’.

**Methods:**

Adolescent mothers (n=1044) from South Africa completed questionnaires between 2017/2019 and 2020/2023, assessing mental health, HIV risks, violence victimisation, education access and hypothesised ‘accelerators’. We used multivariable random effects regression models, adjusting for covariates. Predicted probabilities (marginal effects) were estimated to determine how each identified accelerator, and their combinations, influence the probability of each outcome, offering improved interpretability into the impact of the accelerators.

**Results:**

Three ‘accelerators’ showed protective associations against multiple risks: Food security was associated with reductions in age disparate/transactional sex (OR 0.56, 95% CI (0.42, 0.74)); no contraception use (OR 0.42, 95% CI (0.29, 0.60)); no school enrolment or work engagement (OR 0.46, 95% CI (0.32, 0.67)) and low self-efficacy (OR 0.62, 95% CI (0.46, 0.83)). Non-violent parenting was associated with reductions in suicidality (OR 0.20, 95% CI (0.10, 0.39)); mental health distress (OR 0.44, 95% CI (0.31, 0.64)); transactional/age disparate sex (OR 0.62, 95% CI (0.45, 0.87)); intimate partner violence (OR 0.27, 95% CI (0.14, 0.52)); and sexual violence (OR 0.21, 95% CI (0.10, 0.44)). Respectful clinics were associated with reductions in mental health distress (OR 0.65, 95% CI (0.46, 0.92)); low self-efficacy (OR 0.43, 95% CI (0.33, 0.58)) and condomless sex (OR 0.46, 95% CI (0.35, 0.61). When all three accelerators—compared with none—were provided, risks were greatly lowered. For example, suicidality from 13% to 2%; intimate partner violence from 22% to 5% and sexual violence from 11% to 1%.

**Conclusions:**

This real-world, longitudinal cohort design study identifies services with accelerator impacts that protect adolescent mothers against multiple risks. These can be realised through improved reach of existing services to include adolescent mothers: economic support including government cash transfers, parenting programmes and adolescent-responsive healthcare.

**WHAT IS ALREADY KNOWN ON THIS TOPIC**

- One-third of adolescent girls in Africa are mothers. There is strong evidence of negative impacts on health, education and HIV risks, but almost no evidence of effective interventions.

**WHAT THIS STUDY ADDS**

- This real-world, longitudinal cohort design study shows that adolescent mothers with access to food security, non-violent parenting and clinics where they are treated with respect have improved outcomes across multiple domains.

**HOW THIS STUDY MIGHT AFFECT RESEARCH, PRACTICE OR POLICY**

- Adolescent mothers are a highly vulnerable group, and there is a critical gap in policy and practice regarding how to promote their resilience. These findings demonstrate the potential for a focused set of scalable services to improve a wide range of outcomes for adolescent mothers. The receipt of just three services—social protection, parenting programmes and adolescent health services—offers significant and multifaceted benefits. It supports UN Development Programme-conceptualised theoretical models of ‘accelerator’ interventions, which have multiple simultaneous beneficial impacts. Such findings may be increasingly important to inform services in contexts of reducing aid and fiscal space to support the most vulnerable.

## Introduction

 Worldwide, adolescent parenthood is a marker of health, educational and social disadvantage.^1^,^3^ In Africa, 30% of adolescent girls are already mothers, and rates of adolescent pregnancy increased further during the COVID-19 pandemic.^4^ Early pregnancy is predicted by prior adversities including poverty, orphanhood and abuse,^5^,^9^ both within and outside child marriage.^10^ Adolescent mothers experience a range of negative outcomes, particularly in HIV infection,^11 12^ sexual reproductive health,^13^ intimate partner violence,^14^ mental health distress,^15^,^17^ school dropout and unemployment.^18^ Many of these impacts are intergenerational, for example, children of adolescent mothers experience lower birth weight, reduced educational achievement, lifetime higher poverty and increased risk of becoming adolescent parents themselves.^19 20^

Effective interventions are required to improve outcomes for adolescent mothers and their children. However, reviews highlight a startling lack of services and evidence for this group.^15^,^21^ A systematic review in 2020 found a small number of studies, all in high-income countries. Interventions showed benefits on self-esteem and school attendance, but there was no evidence for effectiveness on health, mental health or violence prevention,^22^ and no effective interventions at all in the Global South. There are a few ongoing small-scale pilots focused specifically on adolescent mothers living with HIV,^23^,^25^ emerging programmatic findings on peer provider models facilitating access to mental health and psychosocial support, and a recent pre–post study in Kenya found improved viral suppression and infant HIV testing after intensive home visitation and case management.^26^

With over 36 million adolescent mothers in the region,^27^ protective provisions need to reach a very large subpopulation. These could be targeted, indicated or delivered at population-level to adolescents. For any approach, in the current context of constrained funding, a feasible strategy may be to identify packages of services that can be delivered within existing systems, are cost-effective and that address multiple needs for adolescent mothers. The UN Development Programme introduced the valuable concept of ‘development accelerators’—actionable interventions, policies, provisions or services that drive significant, cumulative progress across multiple Sustainable Development Goal (SDG) targets, spanning economic, social and environmental dimensions. It is essential to consider why it may be valuable to identify accelerators that impact multiple outcomes rather than viewing SDG targets in isolation, as this reflects underlying theories of the SDGs as interdependent—with development goals profoundly influencing each other.^28 29^ Focussing on improving individual targets via siloed approaches could unintentionally hinder progress for other targets. Online supplemental figure 1 (right) shows four SDG-aligned outcomes which are of utmost importance for adolescent mothers (education, sexual and reproductive health, violence victimisation and mental health). Following recommendations by Nilsson *et al*, each of these targets enables or reinforces the achievement of the others, suggesting the value of accelerator strategies across sectors (see online supplemental figure 1, left).

Previous research used observational data to assess existing access to services and provisions to explore associations with key development outcomes for children and adolescents in South Africa,^30^ and other studies aimed to identify an optimal number of accelerators.^31^ Two studies from South Africa focused on HIV risk behaviours among adolescent mothers, finding multiple impacts of food security,^32^ and another study examined possible impacts of childcare on child and maternal development.^33^ Yet, no study has examined a range of hypothesised accelerators and their potential additive effect for multiple development outcomes among adolescent mothers. We identified a set of hypothesised accelerators to explore their impact on the health and well-being of adolescent mothers, using four approaches. First, we conducted literature reviews of promising approaches for adolescent mothers in the region.^21^ Second, we reviewed observational and interventional evidence on adolescent girls^34 35^ and adult mothers.^36^ Third, we consulted with key agencies representing multiple domains of concern for adolescent mothers: the South African National Department of Basic Education, National Department of Health and National Department of Social Development and UNICEF’s Eastern and Southern Africa Office, UNAIDS, WHO and UNFPA. Fourth, an advisory group of adolescent mothers was involved through participatory techniques^37^ in study conceptualisation, development of measures, identification of variables tested, discussion of initial findings and writing of this paper.^38^

This study tests associations of 11 hypothesised accelerators with health, education and violence risks for adolescent mothers, in a longitudinal cohort of over a 1000 participants in South Africa. It aims to identify a practical and scalable package of accelerator provisions with multiple benefits.

## Methods

### Participants and procedures

We conducted a cohort study of adolescent mothers and their children in urban, periurban and rural South Africa. The Eastern Cape is one of the country’s two poorest provinces^39^ characterised by very high health burden, severe infrastructural challenges and resource constraints.^40 41^ Girls and young women aged 10–24 who had become mothers as adolescents were recruited between 2017 and 2019 (N=1044) and were followed up via telephone interviews starting during the COVID-19 lockdowns in 2020, with all subsequent follow-ups during the second wave conducted remotely. In order to include adolescent mothers who were not accessing services, six parallel sampling strategies were used: recruitment via 73 health facilities, 43 randomly selected quintile 1–3 secondary schools, 9 maternity obstetric units and service provider referrals, door-to-door community recruitment, and snowball sampling by adolescent mothers themselves. All consent forms and questionnaires were available in isiXhosa or English according to participant choice. Wave 2 of the survey took place during the COVID-19 pandemic, and so the interview process was adapted to remote (phone-based) interviews. This required reducing the interview length for feasibility.

Participants chose interview locations, either in or around their own home, or in a space outside their local area. Assisted by trained local interviewers, participants completed two complementary questionnaires about their experiences as an adolescent and as a mother, each taking about 60 min. Participants were encouraged to take as many breaks as they needed during the interview, and it was reiterated that the study can be stopped at any point. Confidentiality concepts were explained to the participants, and confidentiality was maintained throughout the study except where participants requested help or were at risk of significant harm. Over one-third of the study participants were in need of support services; 370 referrals were made throughout waves 1 and 2, including emergency food parcels, assistance with service appointments (eg, applications for ID cards), and connections to counselling. There were no monetary incentives, but all participants received a certificate, refreshments and a participant pack containing useful items selected by our adolescent advisory group (eg, washcloth and soap) and airtime vouchers during the COVID-19 data collection.

We measured hypothesised accelerators, risk factors—all binary and prepiloted with our adolescent advisory group—across adolescent health, education and violence, and sociodemographic covariates.

### Measures

Online supplemental table 1 presents a comprehensive list of all scales and measures. Adolescent mothers’ outcomes were assessed in four categories, all at wave 1 and wave 2, capturing (1) sexual reproductive health and HIV prevention (SDGs 3 and 5); (2) violence victimisation (SDGs 5); mental health (SDG 3) and education (SDG 4). 10 hypothesised protective accelerators were measured at wave 1 and wave 2, except for three accelerator provisions that were not assessed at wave 2 (antenatal care access, positive parenting, mobile health information), due to constraints of remote data collection in COVID-19 lockdowns. Eight sociodemographic covariates were included in all analyses: participants’ age, their age at first pregnancy, the number of children, being the primary caregiver of their child, HIV status, rural/urban residency, orphanhood (maternal or paternal) and household size.

### Statistical analyses

Participants were eligible for inclusion in the analysis if they completed both waves of the data collection. We excluded 274 participants from the analyses because they were unable to be traced at follow-up (76%), refused to participate (18%) or because mother or child had died (6%). The final analyses were based on 771 participants.

First, we examined covariance differences between participants lost to follow-up and those who remained in the study. Second, frequencies and correlations of all measures at each wave of data collection were checked. Third, we examined relationships between all hypothesised accelerators that were measured at two timepoints with all adolescent mother outcomes, using a multivariable random-effects regression model to take into account the repeated-measures nature of the data. Fourth, three hypothesised accelerators which were only available from the baseline wave of data collection (mobile health information, positive parenting and antenatal care) were examined in a lagged longitudinal multivariable regression using outcomes from the follow-up wave, controlling for baseline covariates and outcomes. Fifth, we generated sample weights using a logistic regression containing all baseline covariates and lost to follow-up status as an outcome in order to complete sensitivity analyses for all regression models with and without sample weights. In doing so, we assigned greater weight to participants with characteristics associated with the probability of being lost to follow-up in the weighted regression, which were compared with the ORs to the unweighted analyses. Sixth, to account for the risk of type I error from multiple-hypothesis testing, we adjusted estimated p values using the Benjamini-Hochberg procedure specified with a false discovery rate of 5%.^42^ Finally, we proceeded with all accelerators that showed associations with three or more adolescent mother outcomes, focusing on generating interpretable results. For our models—each calculating binary outcomes—the predicted probabilities/marginal effects are computed (using the postestimation margins command in Stata) to generate probabilities, which can be interpreted as probability changes in the outcome for a one-unit change in the predictor variables. Predicted probabilities allow a percentage improvement by each protective factor, which is derived from the multivariate regression models and, therefore, accounts for demographic and other confounders, preventing the risk of overestimation of effects that can result from reporting of flat percentages. In this analysis, we will calculate the probabilities for each of the outcomes at all possible combinations of the identified (binary) accelerators to assess the effects of all different combinations of these accelerators. Subsequently, we converted these probabilities into percentages of the risk outcome occurring in two scenarios: (1) with receipt of no accelerators and (2) with receipt of all identified accelerators. Calculating probabilities at different combinations of accelerators reflects an underlying assumption that the combined effect of multiple accelerators on the outcome is additive—the sum of their individual effects—and it does not allow for the exploration of more complex interactions.

## Results

Table 1 illustrates covariate differences by lost to follow-up status, showing marginal differences in participants classed as lost to follow-up who were more likely to be living with HIV, living in smaller households and were less likely to receive good parental monitoring.

**Table 1:** Baseline characteristics of study participants by loss to follow-up

	Lost to follow-up		P value
	Yes (N=274) n (%)	No (N=771) n (%)	
Covariates			
Age at interview			
Mean (SD)	18.5 (1.9)	18.3 (1.9)	0.22
Rural location			
Yes	68 (24.8)	231 (30.0)	0.11
Primary caregiver of the child			
Yes	247 (91.1)	714 (93.6)	0.18
HIV status			
Positive	96 (35.0)	218 (28.3)	0.036
Maternal or paternal orphan			
Yes	118 (43.1)	291 (37.7)	0.12
Household size (including participant)			
Mean (SD)	6.0 (2.5)	6.4 (2.7)	0.015
Age at pregnancy with the first child			
Yes	16.8 (1.7)	16.7 (1.7)	0.38
Multiparity			
Yes	28 (10.2)	66 (8.6)	0.41
Hypothesised accelerators			
Food security			
Yes	192 (70.1)	574 (74.4)	0.16
Formal childcare use			
Yes	60 (22.4)	203 (26.8)	0.15
Non-violent parenting			
Yes	245 (89.4)	705 (91.4)	0.32
Parental monitoring			
Yes	94 (34.3)	322 (41.8)	0.030
Positive parenting			
Yes	80 (34.9)	267 (42.2)	0.053
Respectful clinics			
Yes	138 (50.4)	348 (45.1)	0.14
Positive parentingYes	65 (23.7)	185 (24.0)	0.93
Antenatal care Yes	80 (34.9)	267 (42.2)	0.053
Mobile health informationYes	27 (9.9)	85 (11.0)	0.59
Outcome variables			
Condomless sex			
Yes	155 (57.0)	435 (57.5)	0.87
Sex on substances			
Yes	35 (12.8)	75 (9.7)	0.16
No contraception use			
Yes	70 (25.7)	177 (23.1)	0.38
Age disparate (past year) or transactional sex (past year)			
Yes	87 (32.5)	239 (31.6)	0.80
Intimate partner violence			
Yes	21 (9.5)	32 (5.2)	0.025
Sexual violence Yes	3 (1.1)	24 (3.1)	0.071
SuicidalityYes	12 (4.4)	30 (3.9)	0.72
Mental health distress Yes	26 (9.5)	65 (8.4)	0.59
No school enrolment or work engagementYes	123 (50.2)	267 (40.8)	0.012
Low self-efficacy			
Yes	182 (66.24)	469 (60.8)	0.10

### Sample characteristics

Table 2 shows frequencies for each variable for both waves. At wave 1, the mean age of participants was 18.3 years (SD 1.9), and mean age at birth of first child was 16.7 years (SD 1.7). Almost all adolescent mothers were the primary caregiver of their children (93.6%), a third lived in rural communities and 28.3% were living with HIV. About 40% were either maternally or paternally orphaned. At wave 2 of data collection during COVID-19 lockdowns, adolescent mothers reported reductions in food security (p<0.001), less non-violent parenting (p<0.001) and less parental monitoring (p<0.001) while using more childcare (p<0.001) and reporting higher access to respectful clinics (p<0.001).

**Table 2:** Descriptive statistics of all variables across the two time points

	Baseline (N=771)	Follow-up (N=771)	P value
Covariates			
Age at interview	18.3 (1.9)	21.9 (2.0)	<0.001
Rural location (baseline)	231 (30.0)	N/A	N/A
Primary caregiver of the child (baseline)	714 (93.6)	N/A	N/A
Positive HIV status	218 (28.3)	222 (28.8)	0.82
Maternal or paternal orphanhood	291 (37.7)	N/A	N/A
Number of people in household including participant	6.4 (2.7)	N/A	N/A
Age at pregnancy with the first child	16.7 (1.7)	N/A	N/A
Multiparity	66 (8.6)	217 (28.1)	<0.001
Hypothesised accelerators			
Food security (past week)	574 (74.4)	488 (63.4)	<0.001
Formal childcare use (past week)	203 (26.8)	375 (59.4)	<0.001
Non-violent parenting (past year)	705 (91.4)	471 (61.2)	<0.001
Parental monitoring (past 2 months)	322 (41.8)	36 (5.4)	<0.001
Positive parenting (past 2 months)	185 (24.0)	N/A	N/A
Respectful clinics (past year)	348 (45.1)	386 (66.8)	<0.001
Positive parenting (past 2 months)	185 (24.0)	N/A	N/A
Antenatal care (during pregnancy)	267 (42.2)	N/A	N/A
Mobile health information (current)	85 (11.0)	N/A	N/A
School meal (last term of school)	679 (98.5)	681 (88.33)	0.772
Home visitor/social service access (past year)	9 (1.2)	N/A	N/A
Outcome variables			
Condomless sex (last sexual encounter)	435 (57.5)	318 (42.8)	<0.001
Sex on substances (past year)	75 (9.7)	176 (24.6)	<0.001
No contraception use (past year)	177 (23.1)	99 (12.8)	<0.001
Age disparate (past year) or transactional sex (past year)	239 (31.6)	379 (51.2)	<0.001
Intimate partner violence (past year)	27 (5.3)	159 (21.3)	<0.001
Sexual violence (ever)	24 (3.1)	75 (9.8)	<0.001
Suicidality (past month)	30 (3.9)	104 (13.5)	<0.001
Mental health distress (past month)	65 (8.4)	308 (39.9)	<0.001
No school enrolment or work engagement (current)	267 (40.8)	228 (35.9)	0.069
Low self-efficacy (current)	433 (56.2)	552 (71.6)	<0.001

N/A, not available.

Most adolescent mothers’ outcomes had worsened at wave 2, including higher suicidality (p<0.001), increased mental health distress (p<0.001), more age disparate/transactional sex (p<0.001), more sex on substances (p<0.001), more intimate partner violence (p<0.001), more sexual violence (p<0.001) and lower self-efficacy (p<0.001). By contrast, fewer participants reported condomless sex (p<0.007) in addition to more contraception use (p<0.001).

Onlinesupplemental files 2^5^ present correlations between the hypothesised accelerators for the baseline (online supplemental table 2) and follow-up (online supplemental table 3) wave, and correlations between outcomes for the baseline (online supplemental table 4) and follow-up wave (online supplemental table 5).

**Table 3:** Adjusted probabilities (marginal effects) for identified accelerators.

	Adjusted probability	P value of probability difference(reference: no intervention)
Suicidality		
No intervention	13.33 (5.19–21.47)	
Food security alone	12.55 (4.80–20.31)	0.806
Non-violent parenting alone	**3.04 (0.30–5.78**)	**0.002**
Respectful clinics alone	10.14 (3.12–17.16)	0.254
All interventions	**2.07 (0.30–3.83**)	**0.004**
Mental health distress		
No intervention	32.48(25.54 39.41)	
Food security alone	31.62 (24.58–38.65)	0.789
Non-violent parenting alone	**19.25 (14.75–23.75**)	**<0.001**
Respectful clinics alone	**25.32 (18.76–31.88**)	**0.014**
All interventions	**13.62 (10.81–16.44**)	**<0.001**
Age disparate or transactional sex		
No intervention	58.82 (50.59–67.06)	
Food security alone	**44.60 (36.27–52.93**)	**<0.001**
Non-violent parenting alone	**47.62 (41.52–53.73**)	**0.006**
Respectful clinics alone	54.51 (45.53–63.48)	0.177
All interventions	**30.04 (25.99–34.08**)	**<0.001**
Condomless sex		
No intervention	61.96 (53.78–70.14)	
Food security alone	56.58 (48.12–65.04)	0.112
Non-violent parenting alone	65.51 (59.45–71.58)	0.365
Respectful clinics alone	**44.05 (34.99–53.11**)	**<0.001**
All interventions	**42.35 (37.73–46.97**)	**<0.001**
Sex on substances		
No intervention	19.48 (11.79–27.17)	
Food security alone	**13.54 (7.02–20.07**)	**0.031**
Non-violent parenting alone	13.42 (7.99–18.85)	0.058
Respectful clinics alone	19.51 (11.74–27.27)	0.993
All interventions	**9.05 (5.57–12.52**)	**0.012**
No contraception use		
No intervention	29.98 (20.36–39.61)	
Food security alone	**15.39 (8.57–22.22**)	**<0.001**
Non-violent parenting alone	28.12 (21.69–34.55)	0.682
Respectful clinics alone	29.73 (19.59–39.87)	0.939
All interventions	**14.07 (9.99–18.16**)	**0.003**
Intimate partner violence		
No intervention	22.29 (12.89–31.69)	
Food security alone	**14.89 (6.27–23.50**)	**0.044**
Non-violent parenting alone	**7.95 (2.54–13.36**)	**<0.001**
Respectful clinics alone	21.75 (12.15–31.36)	0.896
All interventions	**4.72 (1.10–8.34**)	**<0.001**
Sexual violence		
No intervention	10.73 (1.92–19.54)	
Food security alone	7.83 (0.84–14.81)	0.294
Non-violent parenting alone	**2.55(−0.53–5.64**)	**0.016**
Respectful clinics alone	8.34(.28–16.40)	0.365
All interventions	**1.35(−0.42–3.13**)	**0.020**
No school enrolment or work engagement		
No intervention	48.35 (38.72–57.98)	
Food security alone	**32.71 (23.97–41.44**)	**<0.001**
Non-violent parenting alone	45.60 (38.80–52.40)	0.558
Respectful clinics alone	54.05 (43.77–64.34)	0.112
All interventions	**35.32 (30.31–40.32**)	**0.034**
Low self-efficacy		
No intervention	77.21 (70.49–83.94)	
Food security alone	**68.51 (60.67–76.34**)	**0.002**
Non-violent parenting alone	77.27 (72.19–82.35)	0.986
Respectful clinics alone	**60.38 (51.40–69.36**)	**<0.001**
All interventions	**49.68 (45.14–54.23**)	**<0.001**

Boldface indicates estimates that differ significantly from the reference group (no intervention)

### Associations between hypothesised accelerators and outcomes for adolescent mothers

**Figure 1:**
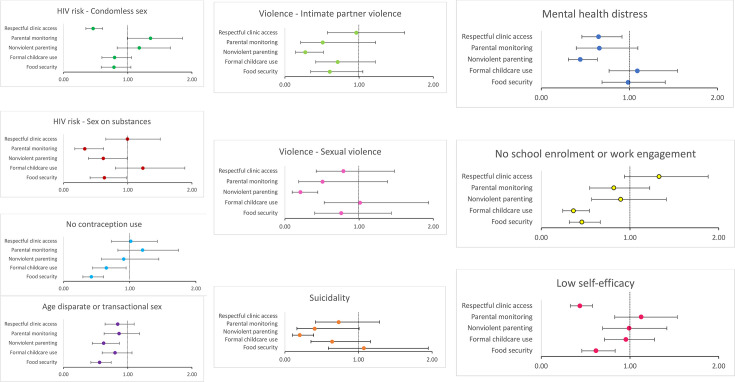
Multivariate associations between all hypothesised accelerators and outcomes for adolescent mothers.

Access to home visitor/social services support was <1.5% and receipt of school meals was 98%, so we were unable to analyse associations for these hypothesised accelerators. Prior to adjustment for multiple comparisons, multivariable models identified five accelerators that were each significantly associated with two or more reduced risks (see figure 1 for an overview), and lagged analyses showed that positive parenting was associated with two reduced risks. After p values were adjusted for multiple comparisons, three accelerators remained significant with benefits on two or more adolescent outcomes. (1) Food security was associated with lower odds of all of: age disparate or transactional sex (p<0.001); no contraception use (p<0.001); no school enrolment or work engagement (p<0.001) and poor self-efficacy (p=0.002); (2) Non-violent parenting was associated with lower odds of all of: suicidality (p<0.001), mental health distress (p<0.001), age disparate/transactional sex (p=0.005); intimate partner violence (p<0.001) and sexual violence (p<0.001); (3) Respectful clinic access was associated with lower odds of all of mental health distress (p=0.014), condomless sex (p<0.001) and low self-efficacy (p<0.001). In addition, two hypothesised accelerator provisions remained significant with one outcome: Childcare access was associated with lower odds of no school enrolment or work (p<0.001), and parental monitoring was associated with lower odds of sex on substances (p=0.001). Please see online supplemental table 6 for a sensitivity analysis comparing these unweighted regression models to weighted models that assign greater weight to participants with higher probability of being lost to follow-up.

**Figure 2:**
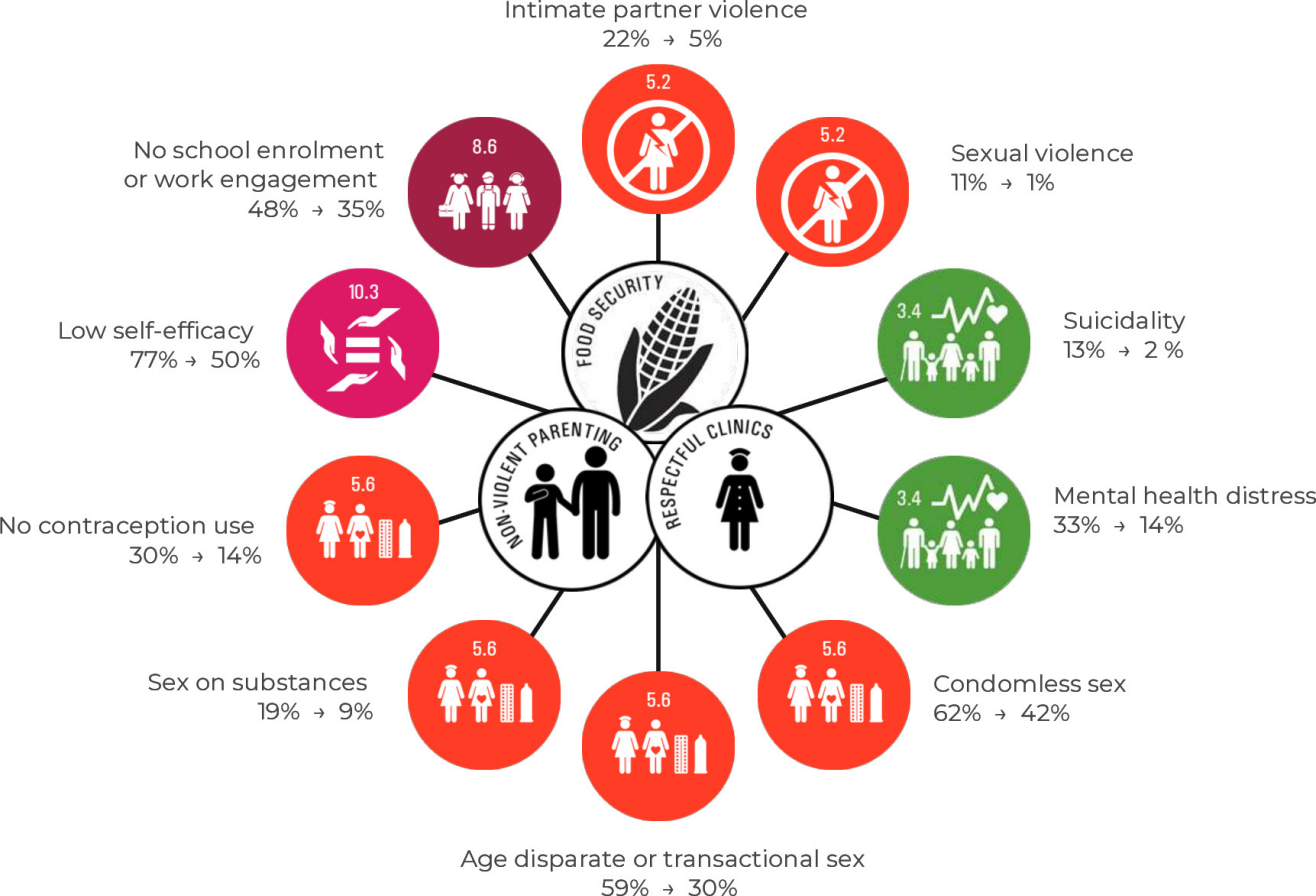
Modelled effects of receipt of no development accelerators and synergy effects of all accelerators.

Table 3 displays the adjusted probabilities (marginal effects) and risk differences for all outcomes for different levels of provisions of the final set of three accelerator provisions. The table illustrates reductions in adjusted probabilities for receipt of a combination of all three provisions (vs none) within the following areas: (1) Sexual and Reproductive Health and Rights and HIV-prevention: Condomless sex showed reductions from 62% to 42%; Sex on substances was reduced from 19% to 9%; No contraception use was reduced from 30% to 14%; (2) Violence prevention: Age disparate/transactional sex was reduced from 59% to 30%; Sexual violence was reduced from 11% to 1%; Intimate partner violence was reduced from 22% to 5%; (3) Mental health: Low self-efficacy was reduced from 77% to 50%; Suicidality was reduced from 13% to 2%; Mental health distress was reduced from 32% to 14% and (4) Education: No school enrolment or work engagement was reduced from 48% to 35% with all three accelerators. All probability differences contrasting adjusted probabilities of reported risk behaviours for the scenarios (1) no provisions and (2) receipt of all three provisions are shown in figure 2.

## Discussion

By 2030, there will be an estimated 160 million adolescent girls in Africa,^43^ and about one-third of these will be mothers.^44^ It is essential to identify scalable services that can support their sexual and reproductive health, HIV-prevention, mental health and education, and in doing so also reduce cycles of intergenerational disadvantage to girls and their children.^45^ This and other studies have shown that adolescent mothers are a vulnerable population which likely experiences amplified stressors during crises. These vulnerabilities need to be considered when developing support structures to increase adolescent mothers’ educational advances, including innovations on how to sustain this support during crises.

This study identifies three ‘accelerator’ provisions that are associated with multiple improved outcomes for adolescent mothers—in a large, longitudinal cohort within a severely resource-constrained South African province: food security, non-violent parenting and respectful clinic access. Food security showed protective associations across four outcomes. Non-violent parenting showed protective associations across five outcomes, and access to respectful clinics across three outcomes. Compared with having access to none of the accelerator provisions, having access to all three accelerators showed substantial improvements on all outcomes. Additionally, childcare access and parental monitoring, respectively, were each protective against one outcome.

All of the identified accelerators have been previously shown to be effectively provided through existing interventions in the African region at national policy or programming level, that could benefit from expansion or adaptation to ensure that they reach adolescent mothers. Food security has been shown to be effectively improved by child-focused government cash transfers^46 47^ as well as by other family-based economic strengthening interventions.^48^ However, these policy programmes are often restricted to women aged >18. Our findings suggest that expanding eligibility to adolescent mothers might have substantial positive impacts, and there may be an additional policy need to remove obstacles to existing sources of support. For instance, adolescent mothers typically do not have direct access to their child’s support grant, which is usually managed by their caregiver or guardian. Recent WHO guidelines recommend parenting programmes for parenting of adolescents as an effective intervention in reducing violent parenting and improving parental monitoring.^49^ These could valuably include adolescent mothers as a target group of adolescents, and also potentially extend to support adolescent parents in parenting for their children. While parenting programmes in Africa had been primarily delivered through Nongovernmental Organizations and bilateral programmes such as PEPFAR, at the 2024 First Ministerial Conference on Ending Violence Against Children, several African governments made national commitments to delivering parenting programmes at scale, working with UNICEF and WHO country offices. Respectful health services are the focus of increasing research, with evidence of effective training programmes^50^,^52^ and potential to extend supportive healthcare to adolescent mothers at both a facility level and in national policies. Childcare is the focus of a World Bank initiative, with a recent review emphasising benefits to labour market participation of mothers.^53^ Extending accessibility of childcare services to adolescent mothers could have meaningful impacts, particularly on their education. The scale of adolescent pregnancy across sub-Saharan Africa requires solutions that can reach large subpopulations, and further research on optimal models for targeted, indicated or population-level delivery is needed. Our research suggests that integrating the identified services into national systems and addressing stigma and accessibility issues are essential for creating a supportive environment for adolescent mothers. These strategies can enhance the effectiveness of parenting programmes and provide holistic support to adolescent mothers, promoting better outcomes for them and their children.

There are also key considerations in designing services that reach adolescent mothers. First, because adolescent motherhood is geographically widely distributed, delivery may be most effective if services are integrated at policy level into existing national health and social welfare systems, such as SRH, antenatal care and postnatal services, national cash transfer programmes and parenting programmes, rather than as standalone targeted services. Second, services should consider avoidance of stigma, as qualitative evidence finds that adolescent mothers experience stigma and shame in community services, related to their poverty (eg, receiving food parcels) or being lone parents, and stigma around adolescent sexual activity.^12 54^ Third, services could be adjusted to increase remote access (such as digitally delivered cash transfers and food vouchers, community health workers), as studies find that adolescent mothers face constraints in travelling to services when caring for an infant or small child.^55^

This study has several limitations. First, the observational study design precludes certainty of causal effects: further research could test packages of care using randomised methods. Second, COVID-19 lockdowns meant that the second wave of data collection could only be collected remotely, and both this method and the pandemic itself may have affected reporting and study outcomes. Third, the present study has not examined the outcomes of the children of the adolescent mothers but will be the focus of future analyses. Fourth, we used a range of self-reported measures in the present study, which could have led to socially desirable answers for some measures (eg, contraception use), and under-reporting on other variables (eg, violence exposures). Fifth, the substantial number of participants classed as lost to follow-up could have introduced selection biases into the analyses. However, our sensitivity analyses demonstrated no significant impact caused by participants who were lost to follow-up. Sixth, the study site was characterised by very limited services, and consequently access to community health and social support/home visits was too low to allow analyses. However, the real-world context is also a key strength of the study, showing that even in a highly deprived context, a set of accelerators was associated with multiple positive outcomes for this highly vulnerable group. Finally, our study aimed to address policy needs by simple, interpretable results. However, these techniques may not fully capture the complexity and directionality of relationships among risk and protective factors, highlighting the need for more nuanced approaches in future research and interventions that consider long-standing and persisting structural inequalities for adolescent mothers, and more broadly in South Africa and the region.

The inequities faced by adolescent mothers are a major issue for gender equity in Africa, population-level HIV-infection rates and child development. This study suggests that a minimal and feasible package of services that already exist in the region—government cash transfers and other economic support, parenting programmes and adolescent-responsive respectful healthcare services—could be extended to reach adolescent mothers with benefits across multiple SDG targets.

## Supplementary material

10.1136/bmjgh-2024-017614 online supplemental file 1

10.1136/bmjgh-2024-017614 online supplemental file 2

10.1136/bmjgh-2024-017614 online supplemental file 3

10.1136/bmjgh-2024-017614 online supplemental file 4

10.1136/bmjgh-2024-017614 online supplemental file 5

10.1136/bmjgh-2024-017614 online supplemental file 6

10.1136/bmjgh-2024-017614 online supplemental file 7

## Data Availability

Data are available on reasonable request.
