# Supplementary material for: Accelerators for improved health among adolescent mothers in South Africa: HIV and violence prevention, sexual reproductive health and education success
Source: BMJ Glob Health. 2025 Jun 2;10(6):e017614. doi: 10.1136/bmjgh-2024-017614 (PMC12142030; doi:10.1136/bmjgh-2024-017614)

Supplementary Figure 1. Conceptual framework for studying for investigating hypothesised accelerators to reduce multiple SDG-aligned risks for adolescent mothers.

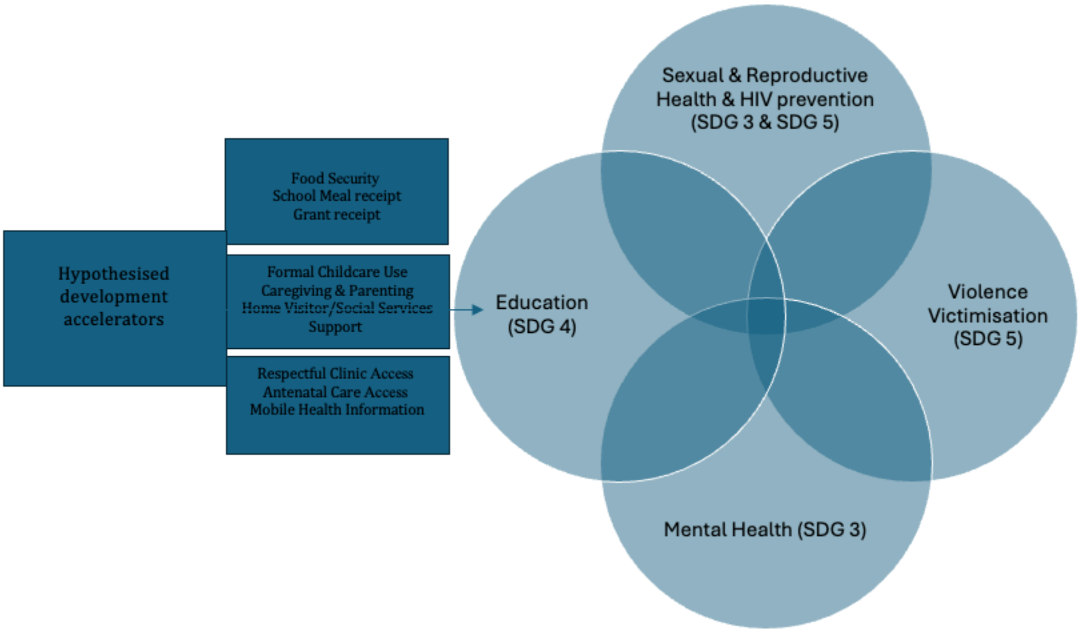

Supplement: online supplemental file 1 [file bmjgh-10-6-s001.pdf]
