# Supplementary material for: Accelerators for improved health among adolescent mothers in South Africa: HIV and violence prevention, sexual reproductive health and education success
Source: BMJ Glob Health. 2025 Jun 2;10(6):e017614. doi: 10.1136/bmjgh-2024-017614 (PMC12142030; doi:10.1136/bmjgh-2024-017614)
Supplement: online supplemental file 2 [file bmjgh-10-6-s002.pdf]

Supplementary Table 1. Overview of scales and measures of the study

Measure	Operationalised measure	Measures in this study	Instrument
**Sustainable Development Goals, Targets, and Indicators aligned with measures for adolescent mothers**			
**5.6 Universal access to sexual and reproductive health**<br><br>**3.3 Reduce number of HIV-infections**	Unsafe sexual practises	**<u>Three self-reported variables based on the following items:</u>**<br>1. Condomless sex *"Did you use condoms the last time you had sex?"*<br>2. Sex on substances *"In the last year, how many times have you had sex when you were drunk or on drugs?"*<br>3. No contraception use *"Which of the following did you and your partner use last year: injection/ implant/ condom, or pill?"*	Items were selected from the PREPARE trial [56] and the African National Survey of HIV and Risk Behavior [57].
**5.2 Eliminate all forms of violence against women and girls**	Experience of sexual, physical, or emotional violence	**<u>Three self-reported variables based on the following items:</u>**<br>1. Age disparate/transactional sex in the past year<br>1.1 "*Think about the oldest person you had sex with in the last year. Was he or she more than 5 years older than you?*<br>1.2 *"Has anyone ever given you a present (money, food, school fees) for having sex?"*<br><br>2. Intimate partner violence in the past year (α = 0.68)<br>2.1 "*I had sex with my partner even when I did not want to, because I was afraid of what they may do*"<br>2.2 "*My boyfriend insulted, swore or said something to spite me*"<br>2.3 "*My boyfriend pushed, shoved, grabbed, or slapped me*"<br><br>3. Sexual violence victimisation over the lifetime<br>3.1 "*Has anyone made you look at their private parts or wanted to look at yours when you did not want to*?"<br>3.2 "*Has anyone touched your private parts, or made you touch theirs, or tried to have sex with you when you did not want to*?"<br>3.3 "*Has anyone had sex with you when you did not want them to*?"	Items were selected from the African National Survey of HIV and Risk Behavior[57]**;** Intimate partner violence was assessed using the revised Conflict Tactics Scale[58] and the WHO Violence against Women Instrument[59]. Self-reported measures of sexual violence included four items from the Juvenile Victimization Questionnaire (JVQ) and two items adapted from previous studies conducted in South Africa among boys and girls [57], [60].

Supplementary Table 1. Overview of scales and measures of the study			
**Measure**	**Operationalised measure**	**Measures in this study**	**Instrument**
**3.4 Promote mental health**	Poor mental health	<u>**Two self-reported variables based on the following items:**</u><br>1. Suicidality in the past month<br>1.1 “*In the past month, did you try to kill yourself*?”<br>1.2 “*In the past month, did you think of a way to kill yourself?”*<br><br>2. Mental health distress covers experiencing symptoms of depression over the past two weeks (α = 0.71), anxiety over the past month (α = 0.86), and posttraumtic stress over the past month (α = 0.85)<br>2.1 *“Nothing will ever work out for me”* (Depression)<br>2.2 *“I am sad all the time”* (Depression)<br>2.3 *“I look ugly”* (Depression)<br>2.4 *“I hate myself”* (Depression)<br>2.5 *“I feel alone all the time”* (Depression)<br>2.6 *“I do everything wrong”* (Depression)<br>2.7 *“I don’t have any friends”* (Depression)<br>2.8 *“I feel like crying every day”* (Depression)<br>2.9 *“Nobody really loves me”* (Depression)<br>2.10 *“Things bother me all the time”* (Depression)<br><br>2.11 *“I worry a lot of the time”* (Anxiety)<br>2.12 *“I worry about what my carers will say to me”* (Anxiety)<br>2.13 *“I feel that others do not like the way I do things”* (Anxiety)<br>2.14 *“It is hard for me to get to sleep at night”* (Anxiety)<br>2.15 *“I worry about what other people think about me”* (Anxiety)<br>2.16 *“I feel alone even when there are people with me”* (Anxiety)<br>2.17 *“I worry about what is going to happen*” (Anxiety)<br>2.18 *“Other children are happier than I am”* (Anxiety)<br>2.19 *“I have bad dreams”* (Anxiety)<br>2.20 *“I wake up scared some of the time”* (Anxiety)<br>2.21 *“I worry when I go to bed at night”* (Anxiety)<br>2.22 *“I am nervous”* (Anxiety)<br>2.23 *“A lot of people are against me”* (Anxiety)<br>2.24 *“I often worry about something bad happening to me”* (Anxiety)<br><br>2.25 *“Do you get upset when you think about what happened?”* (PTSD)	**Suicidality** was assessed via the Mini International Neuropsychiatric Interview for Children and Adolescents (MINI-KID) suicidality and self-harm subscale[61]; **Mental health distress**: Depression (Child Depression Inventory, CDI-S, score ≥3[62]; Patient Health Questionnaire ≥4[63]; **Anxiety** (Children’s Manifest Anxiety Scale—Revised score ≥10; Patient Health Questionnaire ≥4)[64]; **Posttraumatic stress** (Child PTSD checklist, score ≥1 on all four domains: Reexperience, Avoidance, Hyperarousal, and Dysphoria)[65]

Supplementary Table 1. Overview of scales and measures of the study			
**Measure**	**Operationalised measure**	**Measures in this study**	**Instrument**
		2.26 *"When something reminds you of what happened, do you get tense or upset?"* (PTSD)<br>2.27 *"Do you go over and over what happened in your mind?"* (PTSD)<br>2.28 *"Do you think about (or see pictures in your head of) what happened even when you don't want to?"* (PTSD)<br>2.29 *"Do you worry that it might happen again?"* (PTSD)<br>2.30 *"Do you try not to think about what happened?"* (PTSD)<br>2.31 *"Do you try to stay away from things that remind you of what happened?"* (PTSD)<br>2.32 *"Do you make yourself very busy and do things so you won't think about what happened?"* (PTSD)<br>2.33 *"Do you get jumpy or startle easily?"* (PTSD)<br>2.34 *"Do you get annoyed (grouchy) or irritable (kind of angry) really easy?"* (PTSD)<br>2.35 *"Do you feel it's hard to have fun doing things?"* (PTSD)<br>2.36 *"Do you ever feel it's hard to feel happy*?" (PTSD)	
**4.1 All girls and boys complete primary and secondary education**<br><br>**8.6 Promote youth employment, education and training**	No enrolment in school or engagement in work and low self-efficacy	**<u>Two self-reported variables based on the following items:</u>**<br>1. No school enrolment or work engagement<br>1.1 *"Which school do you currently go to?"*<br>1.2 *"Are you currently being paid a wage or salary to work on a regular basis for an employer (that is not yourself), whether full time or part time?"*<br>1.3 *"Have you done any casual work to earn money in the past 30 days?"*<br><br>2. Low self-efficacy (α = 0.88)<br>2.1 *"I can always manage to solve difficult problems if I try hard enough"*<br>2.2 *"I am certain that I can achieve/reach my goals"*<br>2.3 "*I can stay calm because I have ways of solving problems when they come up"*<br>2.4 *"I can handle whatever comes my way"*	Items were developed with a adolescent advisory group[38].

Supplementary Table 1. Overview of scales and measures of the study			
**Measure**	**Operationalised measure**	**Measures in this study**	**Instrument**
**Hypothesised Accelerators**			
**Food Security**		**<u>One self-reported variables based on the following items:</u>**<br>1. Food security *"How many days in the past week (7 days) did you NOT have enough food in your home?"*	South African National Food Consumption Survey[66]
**Formal Childcare Use**		**<u>One self-reported variables based on the following items:</u>**<br>1. Formal childcare use *"How many days in the past week did your child attend daycare/creche?"*	Items were developed with a adolescent advisory group[38]
**Caregiving & Parenting**		**<u>Three self-reported variables based on the following items:</u>**<br>1.Non-violent parenting (physical and emotional α = 0.85):<br>1.1 *"Someone slapped, punched, hit, etc. so that you were hurt/had marks in the last year"*<br>1.2 *"Has someone in home/family ever told you they wished they did not have to look after you?"*<br>1.3 *"Has someone in home/family ever made you feel unwelcome in the home?"*<br>1.4 *"Has someone in family ever said that you would be sent away out of the house?"*<br>1.5 *"Has someone in your home/family ever called you dumb, lazy, or other names?"*<br>1.6 *"Has someone in home/family ever threatened to hurt you?"*<br><br>(2) Parental monitoring (α = 0.93)<br>2.1 *"Your parent or caregiver leaves the house and doesn't tell you where they are going"*<br>2.2 *"You come home from school more than an hour past the time your*	**Non-violent parenting** was assessed using one item on physical abuse from UNICEF's Psychosocial Vulnerability and Resilience Measures For National-Level Monitoring of Vulnerable Children and items UNICEF Psychosocial Vulnerability and Resilience Measures[67]; We measured **parental/caregiver monitoring** and supervision with items from the child form of the Alabama Parenting Questionnaire[68] ; **Positive parenting** was assessed using the Alabama Parenting Questionnaire[68]

Supplementary Table 1. Overview of scales and measures of the study

Measure	Operationalised measure	Measures in this study	Instrument
		parent or caregiver expects you to be home"<br>2.3 *"Your parent or caregiver gets so busy that they forget where you are and what you are doing"*<br>2.4 *"You stay out later than you are supposed to and your caregiver doesn't know it"*<br>2.5 "*You go out without a set time to be home*"<br>2.6 "*You stay out in the evening past the time you are supposed to be at home*"<br>2.7 "Your parent or caregiver does not know who you are friends with"<br><br>(3) Positive parenting (α = 0.91)<br>3.1 *"Your parents or caregiver says you have done something well"*<br>3.2 *"Your parent or caregiver compliments you when you have done something well"*<br>3.3 *"Your parent or caregiver praises you for behaving well"*<br>3.4 *"Your parent or caregiver tells you that they like it when you help out around the house"*<br>3.5 *"Your parent or caregiver rewards or gives something extra to you for behaving well"*<br>3.6 *"Your parents or caregivers hug you or kiss you when you have done something well"*	
**Respectful Clinics Access**		**<u>One self-reported variables based on the following items:</u>**<br>1. Respectful clinics<br>1.1 *"In the past year, I felt my information would be kept safe and confidential"*<br>1.2 "*In the past year, they [clinic staff] got angry and scolded me because of how I take my medication"*<br>1.3 *"In the past year, the clinic/hospital staff were too busy to help"*	Items were developed with a adolescent advisory group [38].

**Supplementary Table 1. Overview of scales and measures of the study**			
**Measure**	**Operationalised measure**	**Measures in this study**	**Instrument**
**Antenatal Care Access**		**One self-reported variables based on the following items:**<br>1. Antenatal care<br>1.1 *"When was your first clinic/hospital appointment when you were pregnant?"*<br>1.2 *"How many pregnancy/antenatal clinic/hospital appointments did you attend during pregnancy?"*	Items - aligned with WHO and South Africa's health ministry recommendations - were developed with a adolescent advisory group [38], [69].
**Mobile Health Information**		**One self-reported variables based on the following items:**<br>1.Mobile health information<br>1.1. *"I use my phone to access health information"*<br>1.2. *"I use my phone to access information about HIV"*	Items were developed with UNICEF and South Africa's Department of Health.
**School Meals**		**One self-reported variables based on the following item:**<br>1. School meals *"In the last term of school, which meals did you have FOR FREE at school?"*	We measured free school meals with one item utilised in a previous study within the same context[70].
**Grant Receipt**		1. Grant receipt *"Are you and your household receiving any grants?"*	Grant receipt was determined based on whether the caregivers reported receiving one or more of the following grants into the home: a retirement pension, state pension, disability grant, child support grant, foster child grant, or care dependency grant.
**Home Visitor/Social Services Support**		1. Home visitor / Social Service Support *"Has anyone visited your home in the past year to help you with your child?"*	Items were developed with a adolescent advisory group[38].

[1][56] C. Mathews *et al.*, 'Effects of PREPARE, a multi-component, school-based HIV and intimate partner violence (IPV) prevention programme on adolescent sexual risk behaviour and IPV: Cluster randomised controlled trial', *AIDS Behav.*, vol. 20, no. 9, pp. 1821–1840, 2016.

1

[57] A. E. Pettifor *et al.*, 'Young people's sexual health in South Africa: HIV prevalence and sexual behaviors from a nationally representative household survey', *AIDS Lond. Engl.*, vol. 19, no. 14, pp. 1525–1534, Sep. 2005, doi: 10.1097/01.aids.0000183129.16830.06.

[58] 'The Revised Conflict Tactics Scales (CTS2): Development and Preliminary Psychometric Data - MURRAY A. STRAUS, SHERRY L. HAMBY, SUE BONEY-McCOY, DAVID B. SUGARMAN, 1996'. Accessed: Feb. 22, 2025. [Online]. Available: https://journals.sagepub.com/doi/10.1177/019251396017003001

[59] 'WHO multi-country study on women's health and domestic violence against women: summary report'. Accessed: Feb. 22, 2025. [Online]. Available: https://www.who.int/publications/i/item/9241593512

[60] D. Finkelhor, S. L. Hamby, R. Ormrod, and H. Turner, 'The Juvenile Victimization Questionnaire: reliability, validity, and national norms', *Child Abuse Negl.*, vol. 29, no. 4, pp. 383–412, Apr. 2005, doi: 10.1016/j.chiabu.2004.11.001.

[61] D. Sheehan *et al.*, 'The validity of the mini international neuropsychiatric interview (MINI)', *Eur. Psychiatry*, vol. 12, no. 5, pp. 232–241, 1997.

[62] M. Kovacs, 'Children's depression inventory', *Acta Paedopsychiatr. Int. J. Child Adolesc. Psychiatry*, 1992.

[63] K. Kroenke, R. L. Spitzer, and J. B. W. Williams, 'The Patient Health Questionnaire-2: validity of a two-item depression screener', *Med. Care*, vol. 41, no. 11, pp. 1284–1292, Nov. 2003, doi: 10.1097/01.MLR.0000093487.78664.3C.

[64] A. B. Gerard and C. R. Reynolds, 'Characteristics and applications of the Revised Children's Manifest Anxiety Scale (RCMAS).', in *The use of psychological testing for treatment planning and outcome assessment*, Lawrence Erlbaum Associates Publishers., 1999, pp. 232–340.

[65] L. Amaya-Jackson, E. Newman, and D. Lipschitz, 'The Child and Adolescent PTSD Checklist in Three Clinical Research Populations', in *Annual Meeting of the American Academy of Child and Adolescent Psychiatry. 2000*, New York.

[66] D. Labadarios *et al.*, 'National food consumption survey in children aged 1-9 years: South Africa 1999', *Forum Nutr.*, vol. 56, pp. 106–109, 2003.

[67] L. Snider and A. Dawes, 'Psychosocial vulnerability and resilience measures for national-level monitoring of orphans and other vulnerable children', Unicef, Cape Town, 2006.

[68] F. Elgar, D. A. Waschbuch, M. R. Dadds, and N. Sigvaldason, 'Development and validation of a short form of the Alabama Parenting Questionnaire', *J. Child Fam. Stud.*, vol. 16, no. 2, pp. 243–259, 2007.

[69] T. M. Hlongwane *et al.*, 'Implementing antenatal care recommendations, South Africa', *Bull. World Health Organ.*, vol. 99, no. 3, pp. 220–227, 2021.

[70] Meinck F, Orkin FM, Cluver L. Does free schooling affect pathways from adverse childhood experiences via mental health distress to HIV risk among adolescent girls in South Africa: a longitudinal moderated pathway model. J Int AIDS Soc. 2019 Mar;22(3):e25262. doi: 10.1002/jia2.25262.
