# Supplementary material for: Accelerators for improved health among adolescent mothers in South Africa: HIV and violence prevention, sexual reproductive health and education success
Source: BMJ Glob Health. 2025 Jun 2;10(6):e017614. doi: 10.1136/bmjgh-2024-017614 (PMC12142030; doi:10.1136/bmjgh-2024-017614)
Supplement: online supplemental file 3 [file bmjgh-10-6-s003.pdf]

Supplementary Table 2. Correlation between hypothesised accelerators, baseline.

	Food security	Formal childcare use	Non-violent parenting	Parental monitoring	Positive parenting	Respectful clinics	Mobile health information	Antenatal care
Food security	1							
Formal childcare use	.01	1						
Non-violent parenting	.10	-.13	1					
Parental monitoring	.27*	-.16*	.32*	1				
Positive parenting	.54*	.01	-.13	.05	1			
Respectful clinics	.34*	.02	-.22*	-.19*	.15*	1		
Mobile health information	.20*	.17	-.24*	-.22*	.08	.38*	1	
Antenatal care	-.27*	.07	.19*	-.14*	-.09	.05	-.03	1

For hypothesized accelerators at baseline, food security was significantly correlated with all other hypothesized accelerators, except non-violent parenting and formal daycare use. In addition, adolescents exposed to higher non-violent parenting from their own caregiver reported receiving higher parental monitoring (r = .32) and access to antenatal care (r = .19). Non-violent parenting was also negatively associated with respectful clinic access (r = -.22) and mobile health information (r = -.24). Receiving higher parental monitoring from their own caregiver was associated with respectful clinics (r = -.19), mobile health information (r = -.22), and antenatal care (r = -.14). Access to respectful clinics was associated with higher use of mobile health information (r = .38) and positive parenting (r = .15).
