# Supplementary material for: Accelerators for improved health among adolescent mothers in South Africa: HIV and violence prevention, sexual reproductive health and education success
Source: BMJ Glob Health. 2025 Jun 2;10(6):e017614. doi: 10.1136/bmjgh-2024-017614 (PMC12142030; doi:10.1136/bmjgh-2024-017614)
Supplement: online supplemental file 4 [file bmjgh-10-6-s004.pdf]

Supplementary Table 3. Correlation between hypothesised accelerators, follow-up.

	Food security	Formal childcare use	Non-violent parenting	Parental monitoring	Clinic
Food security	1				
Formal childcare use	.15	1			
Non-violent parenting	.23*	.04	1		
Parental monitoring	.07	-.07	.26		
Respectful clinics	.36*	.08	.19*	.26	1

At follow-up, only food security was associated with higher reports of adolescents' exposure to non-violent caregiving (r = .23), and respectful clinics use (r = .39). Exposure to non-violent caregiving was also associated with respectful clinics access (r = .19).
