# Supplementary material for: Accelerators for improved health among adolescent mothers in South Africa: HIV and violence prevention, sexual reproductive health and education success
Source: BMJ Glob Health. 2025 Jun 2;10(6):e017614. doi: 10.1136/bmjgh-2024-017614 (PMC12142030; doi:10.1136/bmjgh-2024-017614)
Supplement: online supplemental file 5 [file bmjgh-10-6-s005.pdf]

Supplementary Table 4. Correlation between hypothesised outcomes, baseline.

	Condomless sex	Sex on substances	No contraception use	Age disparate or transactional sex	Intimate partner violence	Sexual violence	Suicidality	Mental health distress	No school enrolment or work engagement	Low self-efficacy
Condomless sex	1									
Sex on substances	.07	1								
No contraception use	.38*	-.07	1							
Age disparate or transactional sex	.08	.18	.24*	1						
Intimate partner violence	-.17	.28	-.26	.11	1					
Sexual violence	-.57*	.37*	-.24	.13	.58*	1				
Suicidality	-.12	.15	-.04	.15	.07	.21	1			
Mental health distress	.02	.14	.16	.10	.04	.22	.54*	1		
No school enrolment or work engagement	-.06	.13	-.07	.13	.14	.25	.14	.17	1	
Low self-efficacy	.46*	.01	.11	.09	-.02	-.24	-.21	.02	.06	1

Table 5 shows that, at baseline, higher suicidality was associated with higher mental health distress (*r* = .54). HIV risk behaviours were correlated, including associations between age disparate or transactional sex and no contraception use (*r* = .24); condomless sex (during the last sexual encounter) was associated with no use of other contraceptive methods (*r* =.38), sexual violence (*r* = - .57), and low self-efficacy (*r* = .30); and sex on substances was associated with sexual violence (*r* = .37). Intimate partner violence and sexual violence and sexual violence were correlated (*r* = .58).
