# Supplementary material for: Accelerators for improved health among adolescent mothers in South Africa: HIV and violence prevention, sexual reproductive health and education success
Source: BMJ Glob Health. 2025 Jun 2;10(6):e017614. doi: 10.1136/bmjgh-2024-017614 (PMC12142030; doi:10.1136/bmjgh-2024-017614)
Supplement: online supplemental file 6 [file bmjgh-10-6-s006.pdf]

Supplementary Table 5. Correlation between hypothesized outcomes, follow-up.

	Condomless sex	Sex on substances	No contraception use	Age disparate or transactional sex	Intimate partner violence	Sexual violence	Suicidality	Mental health distress	No school enrolment or work engagement	Low self-efficacy
Condomless sex	1									
Sex on substances	.19*	1								
No contraception use	.25*	.11	1							
Age disparate or transactional sex	.11	.21*	-.04	1						
Intimate partner violence	.33*	.38*	.12	.20*	1					
Sexual violence	.17	.22*	-.06	.10	.40*	1				
Suicidality	.27*	.29*	.17	.05	.34*	.52*	1			
Mental health distress	.13	.31*	.09	.03	.32*	.35*	.57*	1		
No school enrolment or work engagement	.03	.16*	.10	.07	.22*	.13	.16	.03	1	
Low self-efficacy	.07	.25*	.09	.05	.15	.10	.30*	.24*	.04	1

The vast majority of baseline correlations between outcomes were also found in the follow-up wave. In addition, higher suicidality was associated with condomless sex (r = .27), sex on substances (r = .29), intimate partner violence (r = .34), sexual violence (r = .52), and low self-efficacy (r = .30). Higher mental health distress was associated with sex on substances (r = .31), intimate partner violence (r = .32), sexual violence (r = .35), and low self-efficacy (r = .24). Age disparate or transactional sex was associated with sex on substances (r = .21) and intimate partner violence (r = .20). Sex on substances was also associated with intimate partner violence (r = .38) and no school enrolment or engagement with work (r = .16). Intimate partner violence was associated with no school enrolment or work engagement (r = .22).
