# Supplementary material for: Accelerators for improved health among adolescent mothers in South Africa: HIV and violence prevention, sexual reproductive health and education success
Source: BMJ Glob Health. 2025 Jun 2;10(6):e017614. doi: 10.1136/bmjgh-2024-017614 (PMC12142030; doi:10.1136/bmjgh-2024-017614)
Supplement: online supplemental file 7 [file bmjgh-10-6-s007.pdf]

**Supplementary Table 6:** Unweighted and weighted regression results.

| | Unweighted | | Weighted | |
|---|---|---|---|---|
| | OR [CIs] | z | OR [CIs] | t |
| **Suicidality** | | | | |
| Food security | 0.99 [0.6;1.62] | -0.01 | 1.13 [0.69;1.83] | 0.5 |
| Formal childcare use | 0.69 [0.42;1.12] | -1.47 | 0.72 [0.42;1.23] | -1.18 |
| Non-violent parenting | 0.23 [0.14;0.37]*** | -5.95 | 0.18 [0.11;0.3]*** | -6.74 |
| Parental monitoring | 0.4 [0.17;0.89]* | -2.23 | 0.42 [0.2;0.88]* | -2.27 |
| Respectful clinics | 0.81 [0.5;1.29] | -0.86 | 0.77 [0.49;1.22] | -1.07 |
| Age at interview | 1.19 [1.03;1.36]* | 2.48 | 1.21 [1.06;1.37]** | 2.86 |
| HIV status | 1.37 [0.81;2.32] | 1.2 | 1.26 [0.76;2.08] | 0.9 |
| Rural location | 0.88 [0.52;1.47] | -0.48 | 0.82 [0.5;1.36] | -0.72 |
| Primary caregiver of the child | 0.69 [0.29;1.63] | -0.84 | 0.75 [0.3;1.85] | -0.62 |
| Maternal or paternal orphan | 1.01 [0.63;1.62] | 0.06 | 1.11 [0.68;1.83] | 0.44 |
| Household size | 0.93 [0.85;1.02] | -1.42 | 0.95 [0.88;1.04] | -1.02 |
| Age at pregnancy | 0.71 [0.6;0.84]*** | -3.99 | 0.72 [0.61;0.84]*** | -4.12 |
| Multiparity | 1.33 [0.75;2.35] | 1 | 1.17 [0.63;2.14] | 0.5 |
| | | | | |
| **Mental health distress** | | | | |
| Food security | 0.98 [0.69;1.4] | -0.07 | 1.02 [0.73;1.43] | 0.17 |
| Formal childcare use | 1.07 [0.76;1.51] | 0.41 | 1.11 [0.79;1.55] | 0.61 |
| Non-violent parenting | 0.36 [0.25;0.51]*** | -5.66 | 0.32 [0.22;0.46]*** | -6.09 |
| Parental monitoring | 0.56 [0.34;0.91]* | -2.29 | 0.49 [0.31;0.76]** | -3.15 |
| Respectful clinics | 0.72 [0.52;1.01] | -1.85 | 0.67 [0.48;0.93]* | -2.36 |
| Age at interview | 1.38 [1.25;1.53]*** | 6.41 | 1.34 [1.22;1.47]*** | 6.39 |
| HIV status | 0.88 [0.6;1.28] | -0.66 | 1.02 [0.7;1.48] | 0.13 |
| Rural location | 0.82 [0.57;1.17] | -1.07 | 0.74 [0.53;1.05] | -1.66 |
| Primary caregiver of the child | 0.66 [0.35;1.23] | -1.29 | 0.87 [0.5;1.51] | -0.47 |
| Maternal or paternal orphan | 0.79 [0.56;1.1] | -1.35 | 0.84 [0.61;1.16] | -1.02 |
| Household size | 0.99 [0.93;1.05] | -0.19 | 1.02 [0.96;1.08] | 0.76 |
| Age at pregnancy | 0.83 [0.74;0.93]** | -3 | 0.85 [0.77;0.95]** | -2.81 |
| Multiparity | 1.1 [0.73;1.67] | 0.47 | 1.02 [0.67;1.55] | 0.1 |
| | | | | |
| **Age disparate or transactional sex** | | | | |
| Food security | 0.56 [0.42;0.74]*** | -4.07 | 0.47 [0.36;0.61]*** | -5.48 |
| Formal childcare use | 0.79 [0.59;1.06] | -1.54 | 0.82 [0.62;1.09] | -1.3 |
| Non-violent parenting | 0.61 [0.44;0.84]** | -2.94 | 0.55 [0.4;0.77]*** | -3.48 |
| Parental monitoring | 0.85 [0.62;1.17] | -0.96 | 1.05 [0.79;1.4] | 0.36 |
| Respectful clinics | 0.84 [0.65;1.1] | -1.22 | 0.83 [0.64;1.06] | -1.43 |
| Age at interview | 1.18 [1.09;1.28]*** | 4.04 | 1.2 [1.11;1.3]*** | 4.57 |
| HIV status | 1.2 [0.89;1.62] | 1.2 | 1.27 [0.96;1.67] | 1.7 |
| Rural location | 0.9 [0.68;1.19] | -0.71 | 0.95 [0.74;1.23] | -0.34 |
| Primary caregiver of the child | 1.14 [0.66;1.94] | 0.48 | 1.31 [0.81;2.13] | 1.11 |
| Maternal or paternal orphan | 0.81 [0.62;1.05] | -1.54 | 0.84 [0.66;1.07] | -1.36 |
| Household size | 1 [0.96;1.05] | 0.33 | 1 [0.95;1.05] | 0.17 |
| Age at pregnancy | 0.9 [0.82;0.99]* | -1.96 | 0.89 [0.81;0.98]* | -2.26 |
| Multiparity | 0.93 [0.64;1.36] | -0.33 | 0.9 [0.62;1.31] | -0.51 |

| | | | | |
|---|---|---|---|---|
| **Condomless sex** | | | | |
| Food security | 0.78 [0.59;1.04] | -1.66 | 0.94 [0.72;1.21] | -0.47 |
| Formal childcare use | 0.8 [0.61;1.06] | -1.53 | 0.8 [0.61;1.05] | -1.58 |
| Non-violent parenting | 1.25 [0.9;1.73] | 1.35 | 1.1 [0.81;1.5] | 0.64 |
| Parental monitoring | 1.38 [1.02;1.86]* | 2.13 | 1.32 [1.01;1.74]* | 2.06 |
| Respectful clinics | 0.46 [0.35;0.59]*** | -5.94 | 0.47 [0.37;0.6]*** | -6.08 |
| Age at interview | 0.96 [0.89;1.04] | -0.9 | 0.9 [0.84;0.98]* | -2.41 |
| HIV status | 0.87 [0.64;1.17] | -0.89 | 0.87 [0.66;1.14] | -0.99 |
| Rural location | 1.33 [1.01;1.74]* | 2.07 | 1.43 [1.11;1.84]** | 2.81 |
| Primary caregiver of the child | 1.95 [1.14;3.32]* | 2.45 | 2.11 [1.36;3.27]*** | 3.33 |
| Maternal or paternal orphan | 0.93 [0.72;1.2] | -0.5 | 0.87 [0.69;1.1] | -1.1 |
| Household size | 0.98 [0.94;1.03] | -0.51 | 1 [0.95;1.05] | 0.16 |
| Age at pregnancy | 0.99 [0.9;1.09] | -0.06 | 1.02 [0.93;1.12] | 0.6 |
| Multiparity | 0.71 [0.49;1.04] | -1.73 | 0.74 [0.51;1.07] | -1.55 |
| | | | | |
| **Sex on substances** | | | | |
| Food security | 0.67 [0.46;0.98]* | -2.03 | 0.64 [0.45;0.9]* | -2.5 |
| Formal childcare use | 1.2 [0.82;1.74] | 0.96 | 1.29 [0.89;1.86] | 1.36 |
| Non-violent parenting | 0.63 [0.43;0.94]* | -2.24 | 0.59 [0.4;0.87]** | -2.62 |
| Parental monitoring | 0.34 [0.19;0.62]*** | -3.54 | 0.34 [0.2;0.57]*** | -4.05 |
| Respectful clinics | 0.95 [0.66;1.35] | -0.27 | 0.97 [0.68;1.38] | -0.16 |
| Age at interview | 1.23 [1.11;1.37]*** | 4.03 | 1.24 [1.12;1.37]*** | 4.32 |
| HIV status | 0.93 [0.62;1.4] | -0.31 | 0.96 [0.63;1.46] | -0.16 |
| Rural location | 0.62 [0.42;0.93]* | -2.27 | 0.55 [0.37;0.81]** | -3.02 |
| Primary caregiver of the child | 0.74 [0.38;1.44] | -0.87 | 0.6 [0.34;1.05] | -1.76 |
| Maternal or paternal orphan | 0.89 [0.62;1.28] | -0.6 | 0.87 [0.62;1.22] | -0.77 |
| Household size | 1 [0.94;1.07] | 0.21 | 1.03 [0.97;1.1] | 1.02 |
| Age at pregnancy | 0.88 [0.77;1] | -1.92 | 0.89 [0.78;1.01] | -1.73 |
| Multiparity | 0.75 [0.46;1.22] | -1.14 | 0.74 [0.45;1.2] | -1.2 |
| | | | | |
| **No contraception use** | | | | |
| Food security | 0.43 [0.31;0.6]*** | -4.91 | 0.44 [0.32;0.6]*** | -5.16 |
| Formal childcare use | 0.66 [0.46;0.95]* | -2.23 | 0.66 [0.47;0.94] | -2.28 |
| Non-violent parenting | 0.95 [0.62;1.45] | -0.22 | 1.11 [0.74;1.66] | 0.5 |
| Parental monitoring | 1.18 [0.83;1.68] | 0.95 | 1.26 [0.92;1.73] | 1.5 |
| Respectful clinics | 0.99 [0.72;1.36] | -0.02 | 0.95 [0.72;1.26] | -0.33 |
| Age at interview | 0.79 [0.71;0.88]*** | -4.25 | 0.77 [0.69;0.85]*** | -5.14 |
| HIV status | 1.19 [0.82;1.73] | 0.93 | 1.23 [0.87;1.73] | 1.19 |
| Rural location | 1.09 [0.79;1.52] | 0.55 | 0.99 [0.73;1.34] | -0.05 |
| Primary caregiver of the child | 1.28 [0.64;2.56] | 0.71 | 1.34 [0.68;2.63] | 0.87 |
| Maternal or paternal orphan | 1.09 [0.8;1.48] | 0.55 | 0.99 [0.74;1.31] | -0.04 |
| Household size | 1.01 [0.95;1.06] | 0.44 | 1.02 [0.97;1.07] | 0.81 |
| Age at pregnancy | 1.04 [0.92;1.17] | 0.67 | 1.12 [1;1.25]* | 2.01 |
| Multiparity | 1.73 [1.08;2.76]* | 2.3 | 2.16 [1.4;3.33]*** | 3.49 |
| | | | | |
| **Intimate partner violence** | | | | |
| Food security | 0.66 [0.42;1.03] | -1.8 | 0.69 [0.45;1.05] | -1.7 |
| Formal childcare use | 0.78 [0.49;1.22] | -1.06 | 0.72 [0.46;1.12] | -1.45 |
| Non-violent parenting | 0.27 [0.17;0.42]*** | -5.75 | 0.27 [0.17;0.42]*** | -5.76 |

| | | | | |
|---|---|---|---|---|
| Parental monitoring | 0.56 [0.27;1.17] | -1.52 | 0.63 [0.32;1.24] | -1.31 |
| Respectful clinics | 1.01 [0.65;1.56] | 0.06 | 1.09 [0.71;1.66] | 0.4 |
| Age at interview | 1.24 [1.09;1.41]*** | 3.38 | 1.28 [1.14;1.45]*** | 4.11 |
| HIV status | 1.42 [0.88;2.31] | 1.44 | 1.33 [0.82;2.15] | 1.19 |
| Rural location | 1 [0.63;1.59] | 0.03 | 0.91 [0.58;1.43] | -0.38 |
| Primary caregiver of the child | 1.18 [0.47;2.95] | 0.37 | 1.33 [0.44;4] | 0.51 |
| Maternal or paternal orphan | 1.19 [0.77;1.83] | 0.8 | 1.1 [0.71;1.68] | 0.44 |
| Household size | 0.99 [0.91;1.07] | -0.14 | 1.04 [0.96;1.12] | 0.99 |
| Age at pregnancy | 0.85 [0.73;0.99]* | -1.96 | 0.86 [0.74;1] | -1.9 |
| Multiparity | 0.98 [0.58;1.66] | -0.04 | 0.98 [0.57;1.68] | -0.04 |
| | | | | |
| **Sexual violence** | | | | |
| Food security | 0.76 [0.43;1.34] | -0.92 | 0.77 [0.44;1.36] | -0.88 |
| Formal childcare use | 0.97 [0.55;1.72] | -0.08 | 0.97 [0.56;1.69] | -0.07 |
| Non-violent parenting | 0.23 [0.13;0.41]*** | -4.98 | 0.2 [0.11;0.37]*** | -5.21 |
| Parental monitoring | 0.51 [0.2;1.31] | -1.38 | 0.77 [0.35;1.68] | -0.64 |
| Respectful clinics | 0.83 [0.48;1.44] | -0.62 | 0.8 [0.47;1.37] | -0.8 |
| Age at interview | 1.11 [0.94;1.31] | 1.3 | 1.18 [1;1.39]* | 2.07 |
| HIV status | 1.98 [1.11;3.55]* | 2.31 | 1.71 [0.94;3.11] | 1.77 |
| Rural location | 0.96 [0.53;1.73] | -0.12 | 0.89 [0.47;1.68] | -0.34 |
| Primary caregiver of the child | 1.15 [0.33;3.93] | 0.22 | 1.19 [0.35;3.98] | 0.28 |
| Maternal or paternal orphan | 0.7 [0.39;1.24] | -1.19 | 0.71 [0.4;1.24] | -1.19 |
| Household size | 1.08 [0.99;1.19] | 1.83 | 1.1 [1.01;1.2]* | 2.3 |
| Age at pregnancy | 0.83 [0.69;1] | -1.87 | 0.83 [0.69;0.99] | -2 |
| Multiparity | 1.27 [0.66;2.42] | 0.73 | 1.18 [0.61;2.29] | 0.51 |
| | | | | |
| **No school enrolment or work engagement** | | | | |
| Food security | 0.5 [0.36;0.68]*** | -4.34 | 0.5 [0.37;0.66]*** | -4.75 |
| Formal childcare use | 0.42 [0.3;0.59]*** | -4.98 | 0.38 [0.28;0.52]*** | -5.91 |
| Non-violent parenting | 1.16 [0.8;1.7] | 0.8 | 1.32 [0.92;1.9] | 1.53 |
| Parental monitoring | 0.91 [0.64;1.29] | -0.51 | 0.92 [0.68;1.24] | -0.5 |
| Respectful clinics | 1.15 [0.85;1.55] | 0.95 | 1.14 [0.86;1.51] | 0.95 |
| Age at interview | 1.11 [1.01;1.21]* | 2.3 | 1.12 [1.02;1.22]* | 2.48 |
| HIV status | 3.05 [2.18;4.25]*** | 6.57 | 2.89 [2.08;4]*** | 6.39 |
| Rural location | 0.92 [0.67;1.25] | -0.5 | 0.89 [0.66;1.19] | -0.75 |
| Primary caregiver of the child | 1.89 [0.97;3.69] | 1.87 | 1.99 [1.15;3.46]* | 2.46 |
| Maternal or paternal orphan | 0.98 [0.73;1.32] | -0.08 | 0.98 [0.75;1.29] | -0.08 |
| Household size | 0.93 [0.88;0.98]* | -2.45 | 0.98 [0.92;1.04] | -0.61 |
| Age at pregnancy | 1.08 [0.96;1.2] | 1.39 | 1.11 [0.99;1.24] | 1.92 |
| Multiparity | 1.46 [0.95;2.25] | 1.77 | 1.42 [0.9;2.26] | 1.52 |
| | | | | |
| **Low self-efficacy** | | | | |
| Food security | 0.63 [0.47;0.84]** | -3.11 | 0.61 [0.47;0.8]*** | -3.52 |
| Formal childcare use | 0.91 [0.69;1.21] | -0.59 | 0.87 [0.65;1.16] | -0.92 |
| Non-violent parenting | 0.79 [0.56;1.11] | -1.32 | 0.9 [0.65;1.25] | -0.61 |
| Parental monitoring | 1.01 [0.75;1.36] | 0.09 | 1.05 [0.8;1.38] | 0.4 |
| Respectful clinics | 0.47 [0.36;0.62]*** | -5.52 | 0.44 [0.35;0.57]*** | -6.36 |
| Age at interview | 1.09 [1;1.18]* | 2.13 | 1.09 [1;1.19]* | 2.11 |

| | | | | |
|---|---|---|---|---|
| HIV status | 1.17 [0.86;1.58] | 1.04 | 1.17 [0.88;1.54] | 1.1 |
| Rural location | 1.59 [1.2;2.11]** | 3.28 | 1.7 [1.31;2.2]*** | 4.06 |
| Primary caregiver of the child | 0.49 [0.27;0.88]* | -2.38 | 0.56 [0.31;1] | -1.95 |
| Maternal or paternal orphan | 1.07 [0.83;1.39] | 0.56 | 0.98 [0.77;1.25] | -0.1 |
| Household size | 1.01 [0.97;1.06] | 0.73 | 1.03 [0.98;1.07] | 1.49 |
| Age at pregnancy | 0.88 [0.8;0.97]* | -2.56 | 0.85 [0.78;0.94]** | -3.15 |
| Multiparity | 0.82 [0.56;1.21] | -0.96 | 0.82 [0.56;1.22] | -0.94 |
| | | | | |
